# A systems-based assessment of the PrePex device adverse events active surveillance system in Zimbabwe

**DOI:** 10.1371/journal.pone.0190055

**Published:** 2017-12-22

**Authors:** Paul C. Adamson, Taurayi A. Tafuma, Stephanie M. Davis, Sinokuthemba Xaba, Amy Herman-Roloff

**Affiliations:** 1 School of Medicine, University of California San Francisco, San Francisco, California, United States of America; 2 CDC-Hubert Global Health Fellowship, Division of Scientific Education and Professional Development, Centers for Disease Control and Prevention, Atlanta, Georgia, United States of America; 3 U.S. Centers for Disease Control and Prevention, Harare, Zimbabwe; 4 U.S. Centers for Disease Control and Prevention, Atlanta, Georgia, United States of America; 5 AIDS and TB Programme, Ministry of Health and Child Care, Harare, Zimbabwe; Azienda Ospedaliera Universitaria di Perugia, ITALY

## Abstract

**Background:**

Voluntary Medical Male Circumcision (VMMC) is an effective method for HIV prevention and the World Health Organization (WHO) has recommended its expansion in 14 African countries with a high prevalence of HIV and low prevalence of male circumcision. The WHO has recently pre-qualified the PrePex device, a non-surgical male circumcision device, which reduces procedure time, can increase acceptability of VMMC, and can expand the set of potential provider cadres. The PrePex device was introduced in Zimbabwe as a way to scale-up VMMC services in the country. With the rapid scale-up of the PrePex device, as well as other similar devices, a strong surveillance system to detect adverse events (AE) is needed to monitor the safety profile of these devices. We performed a systems-based evaluation of the PrePex device AE active surveillance system in Zimbabwe.

**Methods:**

The evaluation was based on the Centers for Disease Control and Prevention’s Updated Guidelines for Evaluating Public Health Surveillance Systems. We adapted these guidelines to fit our local context. The evaluation incorporated the review of the standard operating procedures and surveillance system documents. Additionally, structured, in-person interviews were performed with key stakeholders who were users of the surveillance system at various levels. These key stakeholders were from the Ministry of Health, implementing partners, and health facilities in Harare.

**Results:**

Clients were requested to return to the facility for follow-up on days 7, 14 and 49 after placement of the device. In the event of a severe AE, a standard report was generated by the health facility and relayed to the Ministry of Health Child and Care and donor agencies through predefined channels within 24 hours of diagnosis. Clinic staff reported difficulties with the amount of documentation required to follow up with clients and to report AEs. The surveillance system’s acceptability among users interviewed was high, and users were motivated to identify all possible AEs related to this device. The surveillance system was purely paper-based and both duplicate and discrepant reporting forms between sites were identified.

**Conclusion:**

The PrePex AE active surveillance system was well accepted among participants in the health system. However, the amount of documentation which was required to follow-up with patients was a major barrier within the system, and might lead to decreased timeliness and quality of reporting. A passive surveillance system supported by electronic reporting would improve acceptance of the program.

## Introduction

The protective effect of male circumcision (MC) against male acquisition of HIV infection is demonstrated by strong evidence from randomized trials and observational studies [1, 2]. Mathematical models estimate that widespread MC in selected African countries could prevent up to 6 million new HIV infections and 3 million deaths in the next two decades [1, 3]. Consequently, the World Health Organization (WHO) and the Joint United Nations Program on HIV/AIDS (UNAIDS) and other reproductive health organizations promote voluntary male medical circumcision (VMMC) as an HIV prevention intervention, and have issued several guidelines to support the scaling up of MC services [1, 3–5]. However, the challenge implementing countries face is how to safely scale up VMMC services under current resource limitations [2]. The pre-existing surgical methods initially recommended for adult male circumcision require considerable time, trained personnel, and resources, which have limited their expansion.

In response to the need to make VMMC procedures more efficient, the WHO has prequalified male circumcision devices which reduce procedure time, expand the set of potential provider cadres, and increase acceptability of MC [2, 4, 6]. The first device to be prequalified was PrePex, which is a device consisting of an inner plastic ring sitting around the glans and covered by the foreskin, an outer elastic ring compressing the foreskin against the inner ring. This compression constricts blood circulation distally and the distal foreskin becomes necrotic, allowing relatively easy removal one week later. All components were created for single use and disposal [7].

The WHO reviewed clinical data on the safety, efficacy and acceptability of the PrePex device from eight studies conducted in three countries [6, 8]. It was concluded that the device was clinically efficacious in male circumcision and safe for use among healthy men 18 years and older with only 1.7% adverse event (AE) rate [8]. However, outside research settings, a user-friendly and efficient surveillance system must be established to ensure a wider spectrum of adverse events (AEs) are captured with the population-wide introduction of the PrePex device. The WHO developed a guideline on the use of these devices and advised a phased rollout with post-market surveillance, including safety monitoring to identify events that may be rare but serious [6].

Zimbabwe, with a high prevalence of HIV (~15%) and low baseline MC coverage (~9%),[9] is one of the President’s Emergency Plan for AIDS Relief’s (PEPFAR) high-priority countries for VMMC. In Zimbabwe, PEPFAR provides support to the national VMMC program through two implementing partners, which work in collaboration with the Ministry of Health and Child Care (MOHCC). In mid-2014, Zimbabwe’s MoHCC introduced PrePex-based MC for HIV negative males aged 18 years and older as an alternative method to surgical MC. This was done in an effort to increase MC uptake and increase the cohort of MC providers. All countries using PEPFAR funding for PrePex services are required to implement an active adverse events (AE) surveillance system for the first 1,000 clients as the first step of the phased implementation strategy [6]. Active surveillance, which involves reaching out to the source (in this case, clients) to obtain data of interest, provides more reliable data than passive surveillance, which involves receiving reports whenever they are registered [10]. Passive surveillance is less resource-intensive and will be used in the next phase of the PrePex rollout and intended to be a long-term program. Experiences with the active surveillance system is intended to inform the development of the passive surveillance system. Zimbabwe began PrePex rollout and active surveillance in March 2014, utilizing 9 clinical sites, with active surveillance continuing until the first 1,000 clients were completed in September 2014. The passive surveillance phase began in September 2014, is ongoing, and has expanded to 36 clinic sites as of May 2017.

We performed an evaluation of the PrePex AE active surveillance system in Zimbabwe, based on the recommendations put forth in the Centers for Disease Control and Prevention’s (CDC) *Updated Guidelines for Evaluating Public Health Surveillance Systems* [11]. We provide a systems-based assessment of the surveillance system’s attributes and identify strengths and weaknesses of the system. We also make recommendations for improvement of the current PrePex surveillance system, which might be applicable to other countries or health systems looking to scale-up PrePex and for the rollout of VMMC devices in the future.

## Methods

This systems-based evaluation was conducted in February-March 2015 by CDC staff. We performed an assessment of the PrePex active surveillance system for AEs in Zimbabwe using the recommendations put forth in the CDC’s *Updated Guidelines for Evaluating Public Health Surveillance Systems* [11]. All attributes of the surveillance system, with the exception of predictive value positive, were assessed. In order to better understand the structure, design, and various working parts of the surveillance system, the study team reviewed all the survey forms and tools related to this surveillance system, including standard operating procedures, data collection forms, and adverse events reporting forms from the clinics, the implementing partner organizations, MOHCC, and CDC. The review of these items was necessary to provide context and to frame our assessment of the system–for examples, the review allowed us to map the reporting structure and to identify redundancies that were reported by study participants.

We also performed interviews with key stakeholders at various levels within the surveillance system to assess their experiences and perceptions with regard to being users of the surveillance system. Details of specific tasks performed as part of the guidelines are listed in Table 1.

**Table 1:** Adaptations of CDC’s Updated Guidelines for Evaluating Public Health Surveillance Systems for the PrePex adverse events surveillance system evaluation in Zimbabwe.

Tasks in CDC Guidelines	Descriptions and Adaptations of Tasks for Zimbabwe Evaluation
**Task A:** Engage the Stakeholders in the Evaluation	The evaluation was coordinated by CDC-Zimbabwe with input from the MOHCC, USAID, PEPFAR implementing partners–ZACH, ZiCHIRE, I-TECH, and PSI.
**Task B:** Describe the Surveillance System to be Evaluated	Zimbabwe is one of the first countries to scale up PrePex. Zimbabwe’s AE active surveillance system provides an early model for device AE surveillance in a resource limited setting.
**Task C:** Focus Evaluation Design	The objectives were to assess user experiences with the surveillance system at different levels within the surveillance system
**Task D:** Gather Credible Evidence Regarding the Performance of the Surveillance System	We conducted in-person, structured interviews with key informants with various roles within the surveillance system. We reviewed documents pertaining to PrePex active surveillance phases. Our data collection focused on simplicity, data quality, acceptability, sensitivity, timeliness, and stability.
**Tasks E & F:** Justify and State Conclusions, and Make Recommendations; Ensure Use of Evaluation Findings and Share Lessons Learned	The conclusions and recommendations from this evaluation will be shared with key stakeholders at the MOHCC, USAID, and our implementing partners.

*MOHCC–Zimbabwe Ministry of Health and Child Care; USAID–*United States Agency for International Development; *PEPFAR–President’s Emergency Plan for AIDS Relief; ZACH–Zimbabwe Association of Christian Hospitals; ZiChire–Zimbabwe Community Health Research Program; PSI- Population Services International*

Structured interviews with key informants were performed to gather their insight and experiences as users of the surveillance system. These interviews were conducted by Paul Adamson, who was a senior medical student at the time the interviews were performed. The key informants were a convenience-sample from all VMMC implementing partner (IP) organizations, all three health facilities implementing PrePex VMMC in Harare district and the MoHCC head-office. IP and health facilities leadership provided names of the key informants who were directly involved in reporting VMMC data, tracking patients, and coordinating the VMMC program. The study team (PCA and TAT) reached out to these individuals by phone to explain the study purposes and to request a meeting time. Those available during the five-week data collection period were interviewed. Participation in the interviews was voluntary and responses were anonymous.

There were a total of seven interviews performed and all were conducted in-person. Five of the interviews were done with a single individual, while two of the interviews were done in a small group, consisting of three or four members (e.g.–nurse managers, registration clerks, and VMMC nurses). The interviews were done according to a structured interview guide, which included open and close-ended questions (S1 Appendix). During the interviews, careful notes were taken in the context of the interview guide. Interviews were not recorded or transcribed. The interview data were analyzed according to responses to specific questions. They were not coded and we did not use software to analyze themes.

The objectives of the assessment were not to audit or review patient-level data, thus no patient files were reviewed. Aggregate and quantitative data on AE rates, timeliness of reporting, and quality of these data had not been released by the MOHCC and were not available to the study team as part of this assessment.

Each study participant was read a study information sheet before the onset of structured interviews. Verbal consent was obtained and documented by study staff (PA) before the onset of the structured interviews. To protect privacy, names of participants were not recorded as part of the consent process. Non-research determination for this program evaluation was provided by The Medical Research Council of Zimbabwe (MRCZ/A/1879) and the Centers for Disease Control and Prevention IRB (#2014–203).

## Results

All study participants sampled agreed to be interviewed (n = 12). There were four officers from implementing partner organizations (three VMMC Managers and one Monitoring and Evaluation Officer); seven representatives from three health facilities (2 facility managers, 4 nurses and 1 data clerk) and one officer from MoHCC (Monitoring and Evaluation Officer).

### System description

The objective of the MoHCC PrePex active surveillance system was to monitor the safety of VMMC using the PrePex device in routine (non-research/non-experimental) service settings. In order to facilitate tracking of clients, complete locator information including address and cell phone number were captured on standard client intake and tracking forms. Clients were scheduled to return for clinical review on days 7, 14 and 49 after placement of the device. The day 7 review was the day for device removal and clients missing this review day were sent two text messages in the late afternoon that day. If they did not present, up to four phone calls were made on day 8 and if the client failed to present, a home visit was done on day 9 for clinical management and AE documentation. If day 14 and/or 49 reviews were missed, only text messages and phone calls were made but a home visit was not done. All possible AEs were defined by type and classified as mild, moderate or severe, using definitions consistent with the Adverse Event Action Guide for Voluntary Medical Male Circumcision by Surgery with modifications recommended by the template to adapt to device AEs [12]. The system had a total of seven tools (forms and registers) used to report AEs depending on the severity. When reporting a moderate AE for a patient who did not miss his review day, a minimum of four tools would need to be completed, some of which required recording duplicate information.

**Fig 1:**
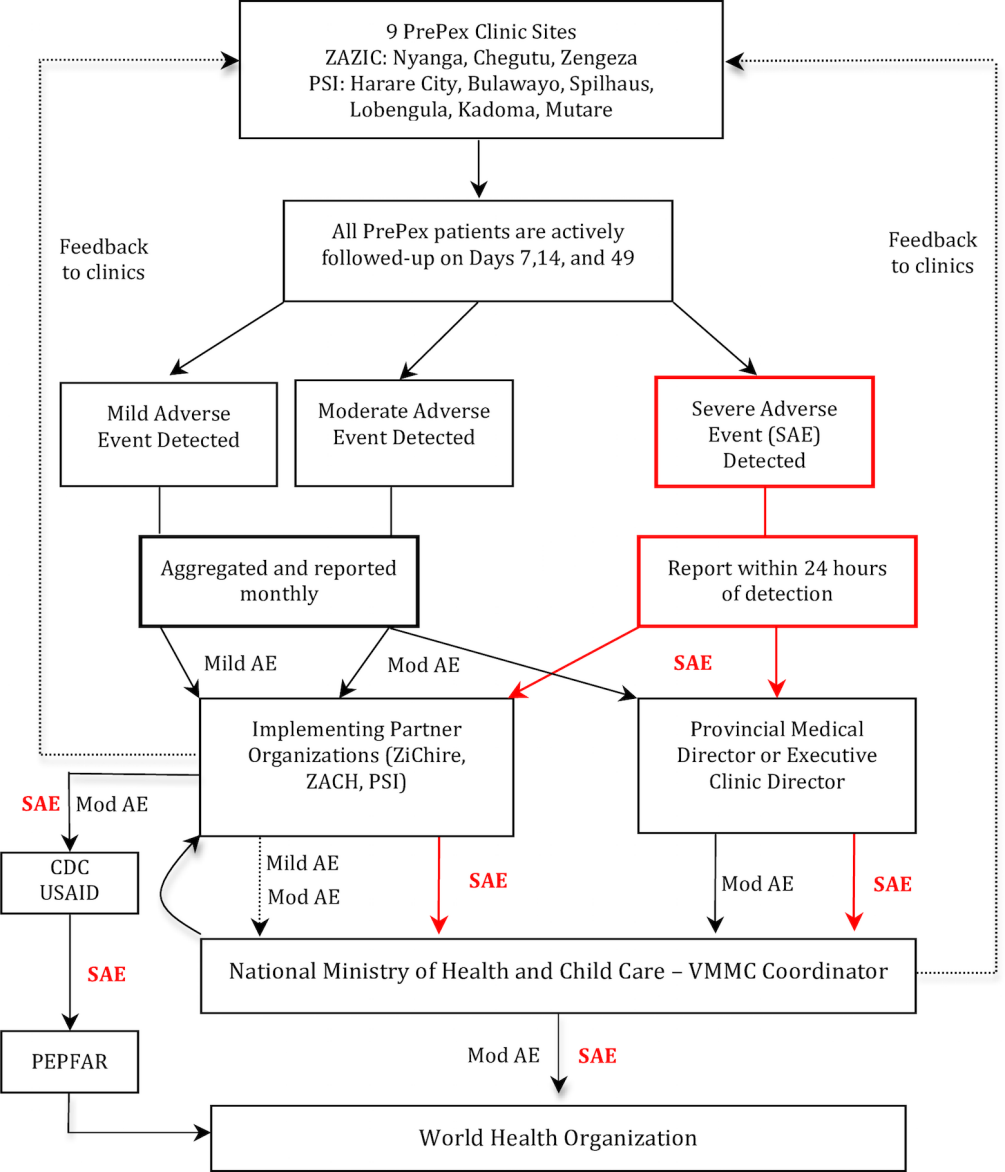
Flow Chart of the PrePex adverse events surveillance reporting system in Zimbabwe.

Reporting channels are shown in a flow sheet (Fig 1). Only moderate and severe AEs were reported to PEPFAR and the WHO; all severities of AEs were reported to the MoHCC. Every month, each site compiled aggregate AE data and the detailed forms for each AE, and submitted a dataset to the implementing partner supporting that site. Dedicated PrePex site managers who were directly employed by partner organizations ensured that sites were collecting the right information and reporting on time. The partner organizations then aggregated data from their supported sites and submitted a dataset to the MoHCC. In the event of a severe AE, a parallel rapid pathway was also used: a standard report was generated by the clinic and relayed to the MoHCC VMMC coordinator and donor agencies (CDC or United States Agency for International Development [USAID]) through predefined channels within 24 hours of diagnosis. MoHCC then compiled a report for WHO while implementing partners reported to PEPFAR. The need for further dissemination to other forums which involve other stakeholders (e.g. quarterly HIV prevention meetings at MoHCC) was highlighted but did not occur.

### Active surveillance system attributes for PrePex device adverse events

#### Simplicity

This reflects both the simplicity of the structure and easy operation of the surveillance system [11]. Study participants from health facilities reported difficulty with the amount of documentation required to follow-up with patients and to report adverse events. For example, if a client does not show-up for review and has a severe AE, five forms including registers would need to be completed. The clinic staff also reported a heavy burden of daily reporting due to the various record-keeping practices; one staff member reported, “there were too many documents and registers to fill out, it impacted our work here at the clinic”.

The MOHCC reported receiving different reporting forms from different clinics, but their content was very similar. The MOHCC needed to contact the IP or clinics to clarify data on duplicate forms. There was confusion about the reporting structure cited by each of the health facilities, with the clinics being unsure about who actually received the follow-up and AE reports. Study participants from health facilities reported that implementing an electronic AE reporting system in the clinics would greatly decrease the burden of reporting. One clinic reported, “it would be simpler to have electronic reporting…we could see all our progress and that of others too, in real-time”.

### Acceptability

This reflects the willingness of persons and organizations to participate in the surveillance system. We asked participants to rate how acceptable the surveillance system was, on a scale from 1–5, with 1 being not at all acceptable and 5 being very acceptable, all health facilities and partners representatives rated the surveillance system a 4 or 5. Participants reported that reporting AEs was important for patient safety and helped to discover new issues associated with the new device. MoHCC was cited to be responsive to suggestions from health facilities; for example, health facilities’ staff requested the client attendance register and this was quickly introduced in the surveillance system. One clinic member reported that “we felt they [IP and MOHCC] listened to our needs.” The existence of dedicated PrePex managers at sites helped to ease the heavy burden of reporting and documenting felt by the health facilities staff. The MoHCC noted difficulty with reporting AE data to WHO, as the surveillance system’s indicators were not those routinely collected electronically by MoHCC, making it necessary to contact implementing partner organizations for the WHO-specific indicators.

One factor that impacted the system’s acceptability was the perceived lack of feedback to the clinical staff. Two clinic staff reported the desire for more reports from the MOHCC or IP regarding the overall progress of the program and feedback regarding AE rates. One clinic site reported, “We never heard anything back from them. We were totally left in the dark with what was happening.” However, another clinic site reported frequent feedback from IP about progress, which improved acceptability.

### Flexibility

A flexible surveillance system is considered to be the one which can adapt to changing information needs or operating conditions with little additional resources. In this surveillance system, any changes to surveillance indicators or to reporting forms could only be done by the MOHCC; individual clinics were not able to make these changes. Very few changes were made during the active surveillance phase; for example, the introduction of an attendance register for organizing data at the clinic and a modification to pain scale reporting. These changes were adopted without incident. One perceived barrier to flexibility was reported by two clinic staff members, who reported that any changes to reporting forms would increase the burden of reporting “once you start doing some types of paperwork, it can become very hard to switch.” The MOHCC reported that changes were handled well, but that this might have been easier, as there were very few clinical sites during the active surveillance phase.

### Representativeness

The active surveillance system was highly representative of early PrePex clients because it encompassed all sites and clients during the initial rollout. The passive surveillance program is currently ongoing and has expanded to 36 sites (current as of May 2017). The 9 sites of active surveillance represent 25% (9/36) of eventual passive surveillance sites. It should also be noted that the sites used during the active surveillance phase were all urban. Therefore, the representativeness of this surveillance system with regard to rural areas, which might have higher risk for AEs related to challenges in transportation, sanitation, and health care access, might be limited.

### Data quality

This typically reflects the completeness and validity of the data recorded, but we did not have access to the actual data and thus had to assess participants’ perceptions of data quality. During active surveillance, all health facilities had a designated person responsible for documentation and reporting. However, participants representing clinics reported difficulties in determining which patients needed follow-up on specific days. Each implementing partner reported conducting at least monthly data audits to ensure proper identification and classification of AEs by clinics. Only one clinic had an AE committee to oversee data quality. All partner organizations reported receiving clear communication about any missing data from clinics. The MoHCC information reporting system had several gaps in the data; for example, receiving duplicated client reporting forms, leading to discrepancies in data, and some reporting forms not highlighting the type of the AE, which required the MOHCC to follow-up with the sites. To de-duplicate the received data, the MOHCC encouraged reporting using a patient’s identification number which was generated during MC registration.

### Sensitivity

For this evaluation, sensitivity referred to the ability to detect AEs, including the ability to monitor changes in the number of cases over time. Participants were asked about measures which were put in place to identify AEs other than reviewing the records. All clinics visited had AE classifications definitions from the MoHCC [12] which they used to assess AEs. However, all clinics visited reported difficulties assessing and reporting pain. Pain reported by MC clients during device placement, removal, and the time in between were used in the classification of AEs. Given the subjective nature of pain, clinic staff reported that using the pain scale might have led to misclassifications of AE. One example that was given at one clinic site was that “one patient said he had 6/10 pain, but was screaming and crying during the device removal, but then another patient reported that he had 9/10 pain with removal, but he was just lying there calmly”.

### Usefulness

This refers to the contribution which this system made to the provision of device based VMMC services. Study participants from all levels of the surveillance system reported that the data collected by this system contributed significantly to the MoHCC’s decision that PrePex was safe and that national scale-up could proceed. Study participants from implementing partners also found the monthly aggregated reporting from clinic sites to be useful, both as a way to monitor progress and to streamline their reporting upwards to CDC/USAID. However, although implementing partner organizations and MoHCC had a record of all reportable AEs, they had not used these for further analyses like assessing the trend in AE rates over the active surveillance period.

### Timeliness

Timeliness reflects the time between steps in this AE surveillance system and more focus was given to severe AEs. All severe AEs were to be reported by the clinics within 24 hours of detection. Although there were cases of partner organizations receiving severe AE report forms more than 24 hours after diagnosis, they did not feel there were problems receiving reports, either by paper or by telephone, from clinics in a timely manner. Study participants from the clinic and IP levels reported that with an electronic reporting system, they would have more timely access to data for the purposes of reviewing and reporting. The MoHCC reported very good performance by the implementing partners with regard to severe AE reporting, but reported some delays with receiving monthly aggregated reports of AEs. The MoHCC acknowledged the need for electronic reporting systems and suggested integrating the reporting system into the District Health Information System 2 (DHIS2), a system that provides high quality aggregate and patient level data elements.

### Stability

This highlights the reliability and consistent availability of the data collected. All health facilities reported that all registers were continually available for reporting. However, in some sites they reported difficulty with phone reception that made it challenging to answer questions related to the reports raised by the Monitoring and Evaluation officers.

## Discussion

We found the study participants, as users of the surveillance system, were motivated to participate in this surveillance system because they wanted to identify all possible AEs related to this device to ensure safety and therefore improve the uptake of VMMC in their communities. Zimbabwe has been cited as a model for maintaining high standards of surgical care during rapid MC scale up [13] and if these users remain motivated, similar standards could be achieved with the PrePex device. Despite the large time burden of reporting, clinic staff were compliant with the documentation that was needed. As noted in other studies [14], listening to individual user feedback by the MoHCC head office staff contributed to the high acceptability of this system.

With regard to the reporting tools, we noted multiple different reporting forms and duplication of some variables between different tools. These can be consolidated and have fewer tools for tracking and reporting AEs. Furthermore, these reporting tools should also be harmonized with WHO reporting indicators, to avoid the need for IP and MOHCC to return to the primary data in order to derive these indicators. Streamlining reporting forms would increase data quality, timeliness, and motivation among surveillance system users.

The existence of dedicated staff to manage data at the site level was believed to have improved the data quality of this system. Although few changes were made, these were smoothly integrated into the system, demonstrating its flexibility. The active surveillance system also benefitted from having a relatively small number of clinics, which likely made it easier to perform staff training and implement these changes. Relying on paper-based reporting has advantages, as was seen in the Sanyati District of Zimbabwe, the presence of all reporting registers at facilities and having paper-based systems contributed to increased stability [15]. However, as the PrePex services are being scaled-up and provided at remote facilities and the surveillance system transitions to passive surveillance, we anticipate challenges in maintaining timeliness of reporting primarily due to the burden of reporting forms and recording duplication.

The development of a streamlined electronic data system, particularly a module embedded within the pre-existing data management system in place at every health facility, was widely desired by key stakeholders in this assessment and could address the issues related to record duplication. Many stakeholders reported desire to integration into DHIS2, while others simply wanted a way to electronically report data. Remote clinics would not be limited by supplies of the required forms, duplicate entries could be avoided, proper routing would be automated, timeliness could be enhanced, and the formatting differences noted between clinics could be eliminated. Allowing implementing partners and MOHCC access to live data might allow easier audits and follow-up on missing data, as well as improve issues around the timeliness of reporting severe AEs and data completeness. While electronic reporting might enhance user experiences and data management, there is not yet evidence to support an electronic data reporting system in resource limited settings.

After completion of active surveillance, the AE surveillance system transitions to passive surveillance, which is similar to the system described here, but without the ongoing outreach and follow-up of clients and without dedicated on-site project managers. The key challenges for the surveillance system, particularly with transition to the passive surveillance phase, are the large time burden on staff, the redundancy of reporting forms, and the ongoing need for training on reporting as the rollout continues and staff change. Challenges in classifying pain were also noted in WHO Technical Advisory Group meetings and these are likely to continue as protocols evolve, particularly because pain perception can be subjective [6]. Maintaining a robust ongoing surveillance system for AE is important as expansion of VMMC services continue. AE surveillance through the channels described here provides funding partners and the WHO with necessary data to monitor safety and to react to serious concerns. For example, surveillance activities by the WHO and PEPFAR during early uptake of VMMC services identified several cases of tetanus, which led to new recommendations regarding vaccinations prior to the procedure, as well as changes to post-procedure care [16–18]. The working group report also notes the need for improving AE surveillance systems and we provide recommendations for improvement here.

There were limitations to this assessment that should be noted. Investigators were limited to participants’ perceptions rather than quantitative system data when assessing quality, timeliness, or sensitivity, and did not assess the predictive value positive attribute. The authors did not have access to quantitative data with respect to the AE reporting forms and timeliness of reports, which limits the conclusions of the study. However, our objectives were to elucidate the experiences of users within the surveillance system and these were addressed. Only urban sites in the Harare district were assessed, which limits the generalizability of this evaluation, particularly to rural or remote areas. The difficulties highlighted in tracking some patients at clinics may have resulted in underreporting of AEs at clinics. Despite assuring confidentiality to the respondents, responses might have been subject to social desirability bias.

## Conclusion

In our evaluation, the PrePex AE surveillance system met its objectives and has provided valuable information in the scale-up of the PrePex device in Zimbabwe and can be a model for other countries and health systems looking to implement a similar surveillance system. Our findings demonstrate the support for an electronic AE reporting system from users at all levels of the surveillance system and we recommend an electronic-based reporting system. Integration with existing electronic reporting tools (e.g.–DHIS2) during the passive surveillance phase might improve reporting, but the feasibility and sustainability of this approach has not been established. MoHCC should take the lead in revising and reducing the number of reporting tools so as to reduce workload on health care workers. It is also imperative to facilitate interactive and iterative dialogue between users at multiple levels of the surveillance system to ensure the system remains highly acceptable and maintains high quality.

## Supporting information

S1 Appendix Example of structured interview guide. (PDF)
