# Supplementary material for: A systems-based assessment of the PrePex device adverse events active surveillance system in Zimbabwe
Source: PLoS One. 2017 Dec 22;12(12):e0190055. doi: 10.1371/journal.pone.0190055 (PMC5741245; doi:10.1371/journal.pone.0190055)
Supplement: S1 Appendix — (PDF) [file pone.0190055.s001.pdf]

Site: ______________________ Date: ______________

Partner Organization: ______________________

Role of person being interviewed: Clinician Nurse Clinic Manager VMMC Staff

Active Surveillance: *The active surveillance component refers to the initial phase of implementation and scale-up of the PrePex device. Active surveillance was in place for the first 1,000 patients undergoing VMMC with PrePex and involved the active follow-up of these patients through 7 weeks after undergoing initial placement of the device.*

Simplicity

What forms did you use to document patient data?

What forms did you use to document patient follow-up data?

How did you know if someone missed their clinic appointment?

What forms did you use to document all AEs at the clinic?

What forms did you use to report AE upwards?

Who are case reports sent to?

Is the AE surveillance system integrated with any other reporting systems at the clinic (surgical VMMC, TB, HIV, etc).

How long does it take to prepare data reports to partners/MOHCC?

Did the number of reporting forms inhibit your ability to perform the surveillance activities in a timely manner?

How much training did it take to prepare you for the active surveillance system?

Flexibility
Were there any changes to the active surveillance system after it was implemented?

How were those changes handled?

Acceptability
How important is it to detect AE to the PrePex device?

How important is it to report those AE due to the partners and the MOHCC?

When you sent reports of your numbers and any AEs, did the reporting entity provide you with any feedback to say they received your reports?

Were the partners or MOHCC responsive to suggestions for improving reporting system?

Did you have any specific suggestions for improving the active surveillance system?

Do clinics receive reports back from partners/MOHCC on AE rates or total numbers?

With regards to filling out active surveillance documentation, conducting follow-up visits, documenting AEs, and reporting AEs, what was the burden on your time?

How easy was it to report AEs upwards?

Were there any costs associated with reporting?

On a scale from 1 to 5, with 1 being not at all acceptable and 5 being extremely acceptable, how acceptable did you find the AE surveillance system to be?

<u>Interview – Clinic Sites</u>

1. Who was in charge of documenting patient data?

2. Who was in charge of documenting follow-up data?

3. Who was in charge of identifying those patients who missed appointments?
    a. Nurse
    b. Clinician
    c. Clinic Manager
    d. VMMC-dedicated staff
    e. Other: ________________
4. How did you know if someone missed a follow-up appointment?

5. How often was the register reviewed to see if patients missed appointments?
    a. Daily - Always
    b. Daily – Usually

c. Some days (e.g- TuTh or MWF)
d. Weekly
e. Other:______________________

6. What is the preferred method by the clinic for getting touch with patients who missed an appointment?
   a. Text
   b. Call
   c. Letter
   d. Home visit
   e. Other: ______________________
7. Who actually conducted the follow-up?
   a. Nurse
   b. Clinician
   c. Clinic Manager
   d. VMMC-dedicated staff
   e. Other: ____________________
8. Has the clinic had to perform any home visits? YES / NO
9. If yes, who conducted the home visit?
   a. Nurse
   b. Clinician
   c. Clinic Manager
   d. VMMC-dedicated staff
   e. Other: _________________
10. Where was home-visit information documented?
   a. Client Tracking Form
   b. Client Intake Form
   c. PrePex Register
   d. Other: ______________________

Sensitivity

Interview

What documents were used to classify an AE? Interviewee should show documentation that was used.

e. AE Definitions from MOH
f. Other?

Who is in charge of reporting AE to MOH and/or partners?

g. Clinician
h. Nurse
i. Clinic Manager
j. VMMC- dedicated staff
k. Other: ______________________

How often were records reviewed to ensure all AEs were captured and recorded?

l. Daily – always
m. Daily – usually
n. Some daily (TuTh or MWF)
o. Weekly
p. Other: ______________________

Did you have difficulty understanding which adverse events needed to be reported upwards? YES / NO

If yes, what was difficult?

i. Ambiguous definition of categories
ii. ???
iii. Other: ______________________

Did the following need to be reported to the directly to MOH during the active surveillance period?

q. Patient presented on Day 6 after placement with a detached device. YES / NO
r. Patient reports 6 out of 10 pain while wearing the device – YES / NO
s. On Day 14, patient is found to have a wound infection that requires IV antibiotics. YES / NO
t. Patient reports 9 out of 10 pain during device removal – YES / NO

Timeliness

Interview

For Clinic Sites:

When someone had severe AE from PrePex, how soon did you report that AE to the MOHCC?

u. Daily – always
v. Daily – usually
w. Some daily (TuTh or MWF)
x. Weekly
y. Other: ______________________

How were AEs reported to MOH?

z. Hard copy
aa. Email
bb. Fax
cc. Phone
dd. Other: __________________________

Similar to the question above, did you have to report to the partners? YES / NO

If YES, how soon?

Daily – always
Daily – usually
Some days (TuTh or MWF)
Weekly
Other: ______________________

When someone had mild or moderate AE from PrePex, how soon did you report that AE to the partners?

a. Daily – always
b. Daily – usually
c. Some days (TuTh or MWF)
d. Weekly
e. Other: ______________________

Were mild/moderate AEs also reported to MOHCC by the clinic? YES / NO

Were there any barriers to reporting these data on time?

Do you have any suggestions for ways to improve the timeliness of reporting AEs?

Stability

Were there any barriers to reporting data upwards? (Power outages, cell phone reception lacking, no phone lines for fax, etc)

Estimate the percentage of time that the system operated fully?

General Questions

What was best part of the active surveillance system – what made it work so well?

What was the hardest part about the active surveillance system?

If you could change one thing about the surveillance system, what would you change?

<u>Passive Surveillance</u>

11. Did you receive any guidance or instructions about changing the way you document and report AEs once the active surveillance phase ended? YES / NO
    a. If yes, who provided this training/instructions?
        i. MOH
        ii. Partner (ZAZIC/PSI)
        iii. Other:____________________
12. Do you understand how the passive surveillance is different from the active surveillance? YES / NO
    a. Can you explain to me the key differences? Do they mention active case follow-up? YES / NO
13. What forms are you using to report AE through the passive surveillance system?
    a. Client Tracking Form
    b. VMMC Review Date Register
    c. Clinic-specific register
    d. Client Intake Form
    e. Other:__________________________
14. Under the passive surveillance program, what does your clinic do if a patient misses the Day 7 removal appointment?
    a. Nothing
    b. Call
    c. Text
    d. Home Visit
    e. Other: __________________________
15. Have you reported any AE to the MOH/partners through the passive surveillance system? YES / NO
    a. If yes, how was the patient identified?
        i. Came back to clinic
        ii. Missed appointment

    iii. Called / texted for follow-up
    iv. Other: ________________________
  b. If yes, what forms did you use to document the SAE?
    i. SAE reporting form
    ii. Client Tracking Form
    iii. VMMC AE register
    iv. Patient's medical chart
    v. Other: ________________________

16. Can you identify some strengths of the <u>passive</u> surveillance system? What makes it work well?

17. What are some weaknesses of the <u>passive</u> surveillance system?

18. If you could change one thing about the <u>passive</u> surveillance system, what would you change?
